# Potential Using of Resveratrol and Its Derivatives in Medicine

**DOI:** 10.5152/eurasianjmed.2024.24392

**Published:** 2024-06-01

**Authors:** Taha Yasin Koc, Selin Dogan, Mehmet Karadayi

**Affiliations:** 1 Institute of Natural and Applied Sciences, Ataturk University, Erzurum, Türkiye; 2 Department of Biology, Ataturk University Faculty of Science, Erzurum, Türkiye

**Keywords:** Piceatannol, resveratrol, resveratrol derivatives

## Abstract

A phytoalexin polyphenolic chemical, resveratrol, can be found in a variety of foods, including cereals, peanuts, grapes, strawberries, and raspberries. It is also known that resveratrol protects the body against cardiovascular diseases as well as various types of cancer. In addition to these health issues, resveratrol is currently the subject of research since it helps treat and prevent a number of illnesses. More clinical research is needed to validate resveratrol’s potential as a therapeutic agent, despite the plethora of in vitro and in vivo evidence to support this. When the literature data are evaluated, the fact that resveratrol has a therapeutic effect in these studies, but it is known to be subject to rapid metabolism despite its low bioavailability and oral absorption of approximately 75%, has directed the studies to resveratrol derivatives, especially piceatannol. Based on recent studies, 4 types of resveratrol derivatives were assessed in this work: hydroxylated compounds, methoxylated compounds, glycosides, and oligomers. Because of their advantageous bioactivities, methoxylated, hydroxylated, and halogenated derivatives have drawn the most interest among these classes. However, as a result of these studies, more studies should be conducted to better understand whether resveratrol derivatives can be recommended as therapeutic agents.

**Main Points**

- Resveratrol is of great interest due to its health benefits.
- The link between the structure and activity of resveratrol is crucial in establishing this compound’s biological effects.
- Resveratrol demonstrates impressive biological activity, including antioxidant, cardioprotective, neurological, and anticancer effects.
- Derivatives of resveratrol also show a broad spectrum of biological actions, such as antibacterial, anti-inflammatory, anti-aging, and anti-cancer properties.

## Introduction

### Polyphenols and resveratrol

**Figure 1.**
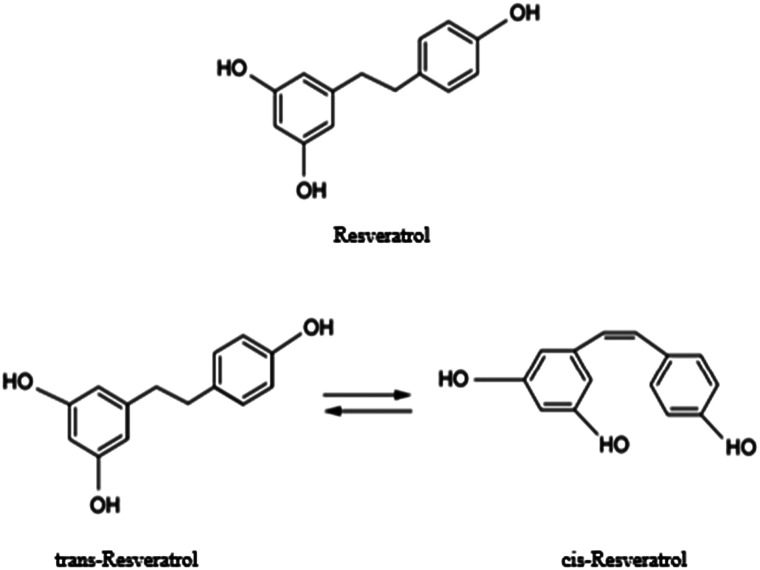
Resveratol and trans-/cis-isomers of resveratrol.

Polyphenols are phytochemicals that are rich in bioactive substances and are primarily found in fruits, vegetables, and soy. Polyphenols are divided into 5 groups: flavonoids, hydrobenzoic acids, hydroxycinnamic acids, lignans, and stilbenes. Over the past few decades, stilbene-based compounds and their variety of biological activities have been the subject of intense research. Of these stilbenes, resveratrol (3,5,40-trihydroxystilbene) in particular is of great interest due to its purported health benefits.^1^ It is reported that in 1940, resveratrol was initially extracted from Veratrum grandiflorum roots.^2^ It was later discovered that, despite its initial protective properties against insect and pathogen attacks, this phytoalexin is a naturally occurring substance that many plants make in reaction to radiation, damage, bacterial or fungal infection, or injury.^3-7^ This naturally occurring phytoalexin chemical has been identified in over 70 different plants, mostly grapes, and is also present in foods consumed by humans.^8^ The molecular structure of resveratrol is made up of 2 aromatic rings joined by a methylene bridge. It can be found in both trans- and cis-isomeric forms (Figure 1).^9^ Plants contain transresveratrol, which is more bioactive than its cis counterpart. The fermentation process of grape skins leads to the formation of cis-resveratrol by the isomerization of resveratrol and the breakdown of resveratrol oligomers. The link between the structure and activity of resveratrol is crucial in establishing this compound’s biological effects.^4,7^

### Effects of resveratrol on human health

The potential of resveratrol in preventing inflammatory diseases was investigated in a recent study.^10^Furthermore, research is being done on the immune-stimulating and antioxidant properties of resveratrol in relation to psoriasis, amyotrophic lateral sclerosis, systemic lupus erythematosus, rheumatoid arthritis, type 1 diabetes, and inflammatory bowel disorders. Additionally, many studies have focused on investigating new treatment options for atopic eczema and psoriasis based on natural compounds such as resveratrol and its derivatives.^7^

Resveratrol modulates cellular processes by acting as an antioxidant. It gets rid of hydroxyl radical, nitric oxide, and superoxide anion.^11^ Cardiovascular disorders, diabetes, cancer, inflammation, aging, and microbial infection have been reported to be prevented or treated by resveratrol.^12-18^

A recent study looked at the impact of resveratrol on skin-related physiological processes in order to assess its significance as an active component in dermatological and cosmetic applications.^19^ In a study conducted by Lin et al,^1^ the use of resveratrol and its derivatives in skin diseases was evaluated. This study makes use of cellular and animal model-based in vitro and in vivo investigations. On the other hand, resveratrol’s limited bioavailability and lack of clinical studies have also been studied, and strategies for overcoming these drawbacks through topical resveratrol application have been explored.^7^

Resveratrol demonstrates impressive biological activity, including antioxidant, cardioprotective, neurological, and anticancer effects. Nevertheless, resveratrol’s limited bioavailability makes it unable to use this compound’s medicinal benefits. Because of this, a lot of research focuses on creating and synthesizing derivatives of this substance in an effort to boost resveratrol’s pharmacological action and bioavailability.^4^ There are numerous resveratrol derivatives in natural sources. There are 4 types of compounds that can be derived from resveratrol: methoxylated compounds, hydroxylated compounds, oligomers, and glycosides. Derivatives of resveratrol also show a broad spectrum of biological actions, such as antibacterial, anti-inflammatory, anti-aging, and anti-cancer properties.^20,21^

**Figure 2.**
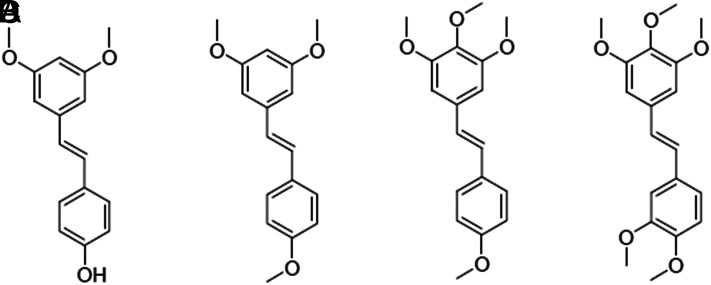
Chemical structures of methoxylated derivatives of resveratrol.^4^ (A) Pterostilbene; (B) trimethoxystilbene; (C) tetramethoxystilbene; (D) pentamethoxystilbene.

**Figure 3.**
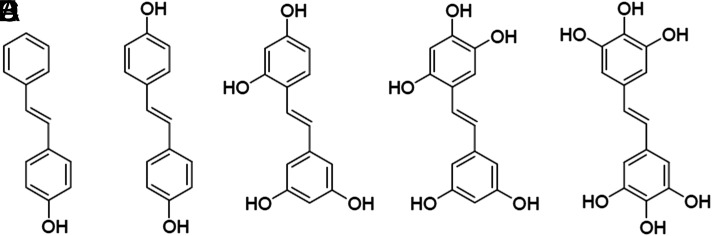
Chemical structures of selected hydroxylated resveratrol derivatives.^4^ (A) Hydroxystilbene; (B) Dihydroxystilbene; (C) Tetrahydroxystilbene; (D) Pentahydroxystilbene; (E) Hexahydrostilbene.

**Figure 4.**
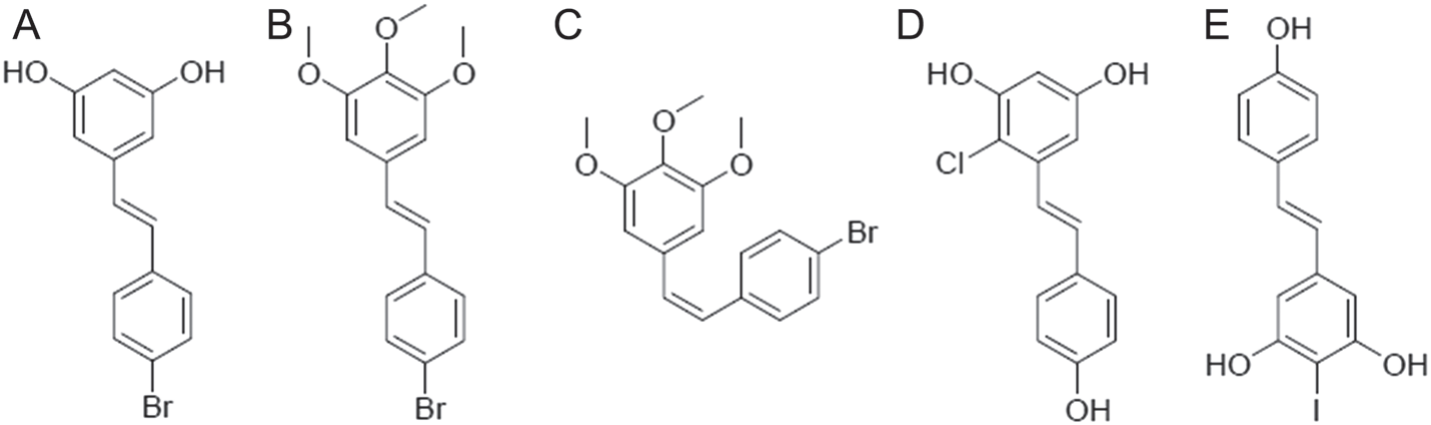
Chemical structures of selected halogenated resveratrol derivatives.^4^ (A) 40-Bromoresveratrol; (B) 3,4,5-trimethoxy-40-bromo-trans-stilbene; (D) 2-chloresveratrol; (E) 4-iodoresveratrol.

Because phase II enzymes metabolize resveratrol, its oral bioavailability is poor, which poses a significant obstacle to its application in the treatment of disease. In addition to having a low bioavailability, resveratrol is highly metabolically active in the bloodstream and has a brief half-life of 8-14 minutes.^22,23^ Previous eras have seen a great deal of research on both natural and synthetic resveratrol derivatives; methoxylated, hydroxylated, and halogenated derivatives in particular have garnered increased interest because of their advantageous bioactivities (Figure 2, Figure 3, Figure 4).^4^

It has been shown in recent studies that adding a hydroxyl group to resveratrol in the ortho position can make it more active than the meta position.^24^ On the other hand, a molecule with more hydroxyl groups has higher antioxidant activity. Furthermore, it is well known that the activity of the molecule can be influenced by the location of these groups and the existence of other subgroups on the aromatic rings.^25^

Pterostilbene has been thoroughly studied as a neuroprotective methoxylated derivative of resveratrol, in which 2 of the 3 hydroxyl groups have been substituted with methoxy groups.^26-28^

### Resveratrol derivatives

Pterostilbene is categorized as a benzylidene compound based on its chemical classification. Pterostilbene’s lipophilicity rises when hydroxyl groups are substituted with methoxy groups, increasing its in vivo bioavailability. Therefore, it causes pterostilbene to show higher biological activity compared to the parent compound resveratrol.^29^ The pharmacological effects of pterostilbene include antioxidant, anticancer, cardioprotective, neuroprotective, and antidiabetic properties.^30^

Pterostilbene restores signaling pathways essential for cell growth and proliferation, shielding human SH-SY5Y neuroblastoma cells from oxidative damage caused by H_2_O_2_.^31^ Another study also showed that estrogen receptor α plays a role in the neuroprotective effects of pterostilbene. This experiment showed that pterostilbene activated Nrf2 signaling and translocated it to the nucleus, protecting against oxidative stress and mitochondrial dysfunction brought on by hyperglycemia using the same cell line.^32^

When pterostilbene was first investigated for its antioxidant potential by Rimando et al,^33^ it was discovered that pterostilbene exhibited free radical scavenging properties that were comparable to those of resveratrol. In a separate study, it was found that resveratrol, pterostilbene, quercetin, and their combinations had the ability to protect erythrocyte membranes from H_2_O_2_-induced lipid peroxidation.^34^ Pterostilbene inhibits signal transduction pathways, including NF-κB expression of vascular endothelial growth factor (matrix metalloproteinase-9 (MMP-9)), MMP-9, and mitogen-activated protein kinase, in order to stop tumor formation in human hepatocellular carcinoma cells. It was reported in another study that it inhibits. Comparably, pterostilbene similarly prevented the growth and death of stomach cancer cells by altering cell cycle-regulating proteins, activating the caspase cascade, and preventing the cells from multiplying, among other mechanisms.^4,35,36^

Despite having the same biological properties as resveratrol, polydatin has a higher bioavailability and a lower enzymatic oxidation risk.^37^ A study was conducted on rats to investigate the metabolism and absorption of polydatin following oral gavage administration at 3 different dosages (50, 100, and 300 mg/kg). In this investigation, it was found that rats’ metabolism and absorption of polydatin were non-linear and dose-dependent.^38^ Comparing polydatin to resveratrol, a study found that when given at the same doses, polydatin’s serum levels were 3-4 times greater than resveratrol’s, suggesting that polydatin might have a faster oral absorption rate than resveratrol.^39^ Resveratrol and polydatin both have anti-inflammatory and antioxidant qualities.^40^

**Figure 5.**
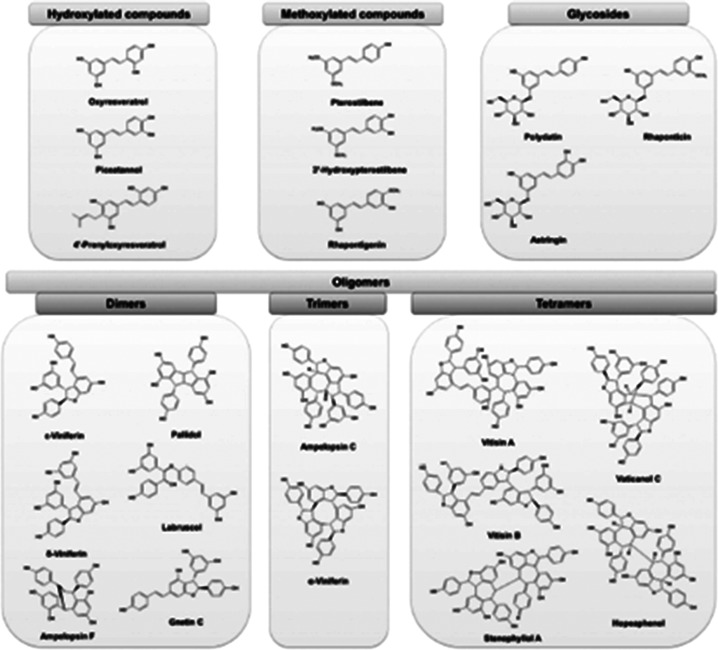
Chemical structures of resveratrol and its naturally occuring derivatives.^1^.

Numerous natural resveratrol derivatives are widely investigated for their bioactivity. These compounds are classified into methoxylated derivatives, hydroxylated derivatives, oligomers, and glycosides based on their structures (Figure 5). The parent compound’s therapeutic flexibility is derived from the hydroxyl group that is added to the resveratrol molecule.^4^ An amazing method to improve resveratrol’s water solubility and therapeutic impact is to add more hydroxyl groups to its phenyl rings. It was discovered that the oral bioavailability of polyhydroxylated derivatives with 3 hydroxyl groups absent from the stilbene moiety was generally low. A study with trans-4,40-dihydroxystilbene, another derivative of resveratrol, showed that this compound was slowly absorbed orally and had low bioavailability in rats.^41^ Studies have shown that molecules containing four hydroxyl groups, such as piceatannol and oxyresveratrol, have better water solubility, quicker absorption, and bioavailability than resveratrol.^42,43^

Many plants are sources of oxyresveratrol that possess antioxidant and anti-inflammatory potential.^44,45^ A recent study has demonstrated that oxyresveratrol is more effective at inhibiting tyrosine oxidation catalyzed by tyrosinase (IC50 = 53 mM) than its parent compound (IC50 >100 mM).^46^ However, another study showed anti-HSV activity in oxyresveratrol. Chuanasa and colleagues^47^ investigated the therapeutic value of oxyresveratrol applied topically for cutaneous HSV infection, as seen in Balb/c mice. When administered for 3 or 6 hours, oxyresveratrol (50 mg/mL) has been shown to elicit 26% and 33% viral inhibition in infected vero cells, respectively.

One of the most studied hydroxylated resveratrol derivatives is piceatannol. Despite its similarity with resveratrol in its molecular structure, piceatannol has been shown in recent studies to be more effective than resveratrol and to have beneficial health properties.^12,18,48-53^

It has also been stated in a recent study that piceatannol can strongly inhibit *Toxoplasma gondii*, the causative agent of toxoplasmosis, which infects the nucleated cells of warm-blooded animals and is known as a zoonotic disease. Piceatannol may therefore be a significant medication contender for the treatment of toxoplasmosis, according to this study’s predictions. In addition, as a result of in vivo studies, it was determined that piceatannol was not toxic to cells within the therapeutic concentration range, in addition to reducing parasite virulence in mice.^54^

Piceatannol possesses several biological activities, including as antiproliferative, chemoprotective, and anti-adipogenic qualities, in addition to lowering inflammation. Furthermore, as evidenced by in vitro and in vivo investigations, piceatannol has been found to have the ability to treat rheumatoid arthritis. Furthermore, it has been demonstrated to reduce osteosarcoma cell proliferation and cause apoptosis in these cells. Because of this, it is believed that piceatannol may be utilized to treat rheumatoid arthritis as a novel anti-inflammatory drug.^18,50-53,55-57^

The effects of piceatannol on mice’s D-galactose-induced aging were examined in a study.^58^ The results of the study showed that piceatannol therapy prevented neuron loss, reduced oxidative stress, increased cell proliferation in the hippocampus and cortex, and maintained spontaneous motor function. It also improved spatial learning and memory abilities. Piceatannol has been found to protect against glutamate excitotoxicity by activating Nrf-2 to induce HO-1 expression in hippocampal neuron cells.^37^

**Figure 6.**
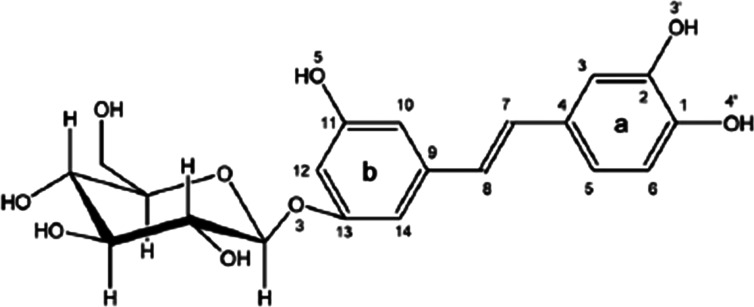
Molecular structure of trans-astringin.

Additionally, the potential of trans-astringin (Figure 6) as a radical scavenger was examined. The study’s findings showed that trans-astringin has significantly higher antioxidant activity than trans-resveratrol.^59^ Compared to resveratrol, astringin’s scavenging effect and antioxidative properties were substantially improved by the presence of a complementary OH group in the B ring (catechol structure).^60^

The effects of resveratrol’s other derivative were seen in rhapontigenin, which has anticancer, anti-inflammatory, cardioprotective, antiallergic, and antithrombotic^61,62^ properties, while isorhapontigenin (3-methoxyresveratrol) has anticancer;^63^ antioxidative;^64^ and anti-inflammatory properties as well. It has been revealed that the oral bioavailability of the isorhapontigenin compound is much higher compared to resveratrol. Because of their diverse biological functions, it is noteworthy that resveratrol derivatives are extremely important.^5^

## Conclusion

Resveratrol’s low solubility, low bioavailability, and potential negative effects make it difficult to use in clinical settings. Resveratrol may have a low bioavailability due to its fast metabolism and limited gastrointestinal absorption. High resveratrol intake might have harmful side effects such as headache, tiredness, and skin rash. Topical resveratrol administration is a viable method to mitigate low oral bioavailability and potential side effects. However, the fact that the resveratrol derivatives evaluated in this study have more bioavailability than resveratrol, have fewer side effects, and have higher solubility directs studies towards resveratrol derivatives. Apart from their anti-aging qualities, resveratrol derivatives have been found to have enhanced bioactivity in comparison to their parent chemical. These properties include anti-inflammatory, anti-cancer, and the ability to eradicate skin problems. There is currently little clinical research on resveratrol, despite the fact that various products have been created for testing in animal and cell-based investigations. The high cost of conducting clinical trials and the need to identify and investigate unknown side effects before testing it clinically may be a factor in this. Further clinical research is needed to support the usage of resveratrol and its naturally occurring derivatives in the future.
